# Comparison of the Pathway to the Inner Ear Between Postauricular and Intramuscular Injection of Dexamethasone in Guinea Pigs

**DOI:** 10.3389/fneur.2022.811626

**Published:** 2022-03-04

**Authors:** Aiping Chen, Wenwen Liu, Lei Xu, Zhiqiang Hou, Zhaomin Fan, Haibo Wang, Mingming Wang

**Affiliations:** ^1^Department of Otorhinolaryngology Head and Neck Surgery, Shandong Provincial ENT Hospital, Cheeloo College of Medicine, Shandong University, Jinan, China; ^2^Shandong Institute of Otolaryngology, Shandong Provincial ENT Hospital, Cheeloo College of Medicine, Shandong University, Jinan, China

**Keywords:** inner ear, drug delivery, *in vivo* optical imaging, dexamethasone, local injection

## Abstract

**Background:**

Postauricular injection as a local therapy has been confirmed to be effective for inner ear diseases. However, the mechanism for the drugs entering the inner ears remains unknown. This study aims to compare the distribution of dexamethasone by intramuscular injection with that by postauricular injection, and explore the pathway of the drugs entering the inner ears.

**Methods:**

An *in vivo* optical imaging system was used to conduct a time course observation to compare the distribution of dexamethasone by intramuscular injection with that by postauricular injection in male guinea pigs. The drug availability in the tympanic mucosa, tympanum, endolymphatic sac, and cochlea was observed by a confocal laser scanning microscope.

**Results:**

The local fluorescent intensity by postauricular injection was significantly higher in the inner ears, and lower in partial peripheral organs, than that by the intramuscular injection. The drug metabolism by postauricular injection exhibited an obviously sustained release effect in the inner ears. Drugs by postauricular injection might enter the endolymphatic sac through the posterior auricular artery and occipital artery, as well as the connections of the mastoid emissary vein, sigmoid sinus and endolymphatic sac.

**Conclusion:**

More drugs concentrated in the inner ear for longer therapeutic time and less systemic delivery implied more effective and less risk of side effects through postauricular injection than intramuscular injection safer for the treatment of inner ear diseases.

## Introduction

Inner ear disorders, especially sensorineural hearing loss (SSNHL), are common diseases in clinical practice. Although various gene therapy targeting several factors have been recently proposed to possess potential therapeutic value, glucocorticoid treatment nevertheless plays an important role in the therapies for these diseases (1, 2). Systemic and intratympanic administrations are known as the most common feasible and achievable routes of drug delivery, but neither of them is optimal. For the systemic steroid administration, the blood-labyrinth barrier blocks the drugs from reaching the endolymphatic fluid of the inner ear (3). High systemic doses of steroid are needed to maintain the concentration level within the inner ear, which could result in undesirable side effects. Local drug delivery to the inner ear *via* intratympanic injection was firstly described for the treatment of Ménière's disease in 1956 (4), and it was then reported that intratympanic injection of methylprednisolone could significantly improve the hearing of patients with SSNHL (5). But intratympanic injection was an invasive procedure which might cause pain, infection, vertigo, and tympanic membrane perforation (6). Moreover, it's also difficult to maintain the concentration level in the inner ear due to drug loss through the eustachian tube. In recent years, intracochlear delivery has been a hot topic as it provides direct access to cochlear cells and the drug dosage can be precisely controlled. However, this innovative method may cause greater risks of surgical trauma and biofouling for indwelling devices, as a result of tissue growth and protein build-up in the scala tympani (7). Therefore, an optimal route for local drug delivery to the inner ear is needed.

Postauricular injection was previously used for local anesthesia in minor otological surgery. Current studies confirmed that glucocorticoid administrated by postauricular injection could result in improved clinical outcomes in the treatment of sudden hearing loss (8). Perilymph pharmacokinetics in animals with postauricular injection showed a longer time to reach peak signal intensity, longer elimination half-life and mean residence time, as well as a greater area under the signal-time curve, than to those with systemic delivery (9). Despite studies measuring drug levels in perilymph (10, 11), it was technically challenging to monitor the pharmacokinetics of administered drugs continuously and to detect the route of entering the perilymph because of the anatomical and structural limitations of the inner ear. And the drug distribution in the inner ear with postauricular injection is still unclear.

In the current study, we used an *in vivo* optical imaging system to evaluate pharmacokinetic changes over time, combining confocal laser scanning microscopy to measure the drug distribution in guinea pigs after either postauricular or intramuscular injections. By comparing the drug (labeled by Sulfo-Cyanine5 carboxylic acid) delivery and distribution through systemic or local administration route alone, the findings would provide a rational support for the use of postauricular injection for the therapy of inner ear diseases in clinical practice.

## Methods

### Animals and Groups

Male guinea pigs weighing 300 ~ 400 g were purchased from Experimental Animal Center, Shandong University, and had free access to food and water. Animal handling procedures were performed in accordance with the National Guide for the Care and Use of Laboratory Animals and approved by the Animal Care and Use Committee of Shandong Institute of Otolaryngology, Shandong Provincial ENT Hospital.

Guinea pigs were allocated into two groups based on the delivery method: (1) dexamethasone labeled by Sulfo-Cyanine5 carboxylic acid (Cy5-dex) postauricular injection group (*n* = 15), (2) Cy5-dex intramuscular injection group (*n* = 15). To compare the pharmacokinetics, they were further divided into subgroups based on the object of detection: drug metabolism by time course observation for 120 h (*n* = 5), drug distribution by fluorescence intensity at 8 h post injection observation (*n* = 5), tissue membrane observation (*n* = 5).

### Drug Delivery

Each animal was administered with Cy5-dex *via* either postauricular or intramuscular injection in a dose of 0.5 ml (0.5 mg/ml). Guinea pigs in the postauricular group were injected in the middle of the right retroauricular groove, by releasing the Cy5-dex solution into the postauricular space between the mastoid process and the posterior wall of acoustic meatus. Animals in the intramuscular group were injected with Cy5-dex solution in the right gluteus maximus. All animals were anesthetized with chloral hydrate (10%, 3 mg/kg; Zhongke, Beijing, China) *via* intraperitoneal injection prior to Cy5-dex delivery, and maintained under anesthesia with inhalational isoflurane during photographing. Core body temperature was maintained at 38°C by a thermistor-controlled heating blanket.

### Bioluminescence Imaging

Dynamic changes in local drug metabolism were evaluated by 120 h of time course using an *in vivo* optical imaging system (Xenogen IVIS Spectrum Imaging System, Caliper, America). Bioluminescent images were captured at 0.5, 1, 2, 4, 8, 12, 24, 48, 72, 96, and 120 h after postauricular or intramuscular injection of Cy5-dex, respectively. Five guinea pigs from each group were decapitated at 8 h post-injection to measure the fluorescence intensity of drug distribution in separated scalp, parietal bone, cerebrum, temporal bone and internal organs by an *in vivo* optical imaging system.

Recorded images were analyzed with Living Image Software (Xenogen IVIS Spectrum Imaging System, Caliper, America). Fluorescent signal data from all tissues were obtained considering the mean value between the anterior and the posterior views. The optical fluorescence intensity was expressed as photon flux (photon count), in units of photon/s/cm^2^/steradian. Each image was displayed as a pseudo-colored and photon-counted image superimposed onto a grayscale anatomic image. To quantify the measured light, we defined regions of interest (ROIs) and examined all values in the same ROI.

### Immunofluorescence

Five guinea pigs in each group were injected with 0.5 ml Cy5-dex per day for three consecutive days. The animals were decapitated on the 4th day, and the parietal bone and brain were removed immediately. The whole skull was fixed in 4% paraformaldehyde for 1 h followed by phosphate-buffered saline (PBS) washing. Tissue stretches from the tympanic mucosa, tympanum and endolymphatic sac were harvested and washed in PBS for three times, adhered to cover slips with cell-tac glue, and then stained with 1:500 DAPI for 30 min to visualize the nuclei of cells. The inner ear was removed and fixed with 4% paraformaldehyde for 4 h, and then demineralized with 4% EDTA for 2 h. The cochlea shell was removed to expose the cochlea basilar membrane, which was then harvested and washed in PBS, adhered to cover slips with cell-tac glue, and stained with 1:500 DAPI and 1:200 phalloidin for 30 min to visualize the hair cell wall.

Tissue stretches were visualized with an inverted DMI 400CS confocal laser scanning microscope (Leica, Germany). All images were collected under the same imaging parameters (magnification 200 ×, scanning space 2 nm). The clearest section was selected and the red fluorescence was outlined with a square for semi-quantification using the Metamorph software (Universal Imaging Corporation). The software reported the intensity as a grayscale value.

### Statistical Analysis

The mean fluorescence intensity of each group was calculated, and presented as mean ± standard deviation. *T*-test was used for comparisons of data between different groups. *P* < 0.05 was considered statistically significant. All statistical analyses were performed with SPSS v. 17.0 software program (SPSS Ltd, Chicago, Illinois, USA).

## Results

### Bioluminescence Imaging

**Figure 1:**
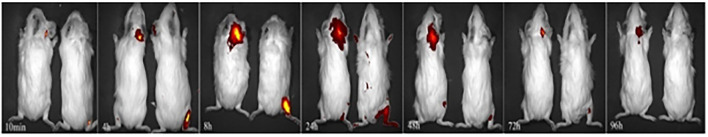
Dynamic changes in local drug metabolism. The guinea pigs on the left side in each figure are from the postauricular injection group and those on the right side are from the intramuscular injection group.

**Figure 2:**
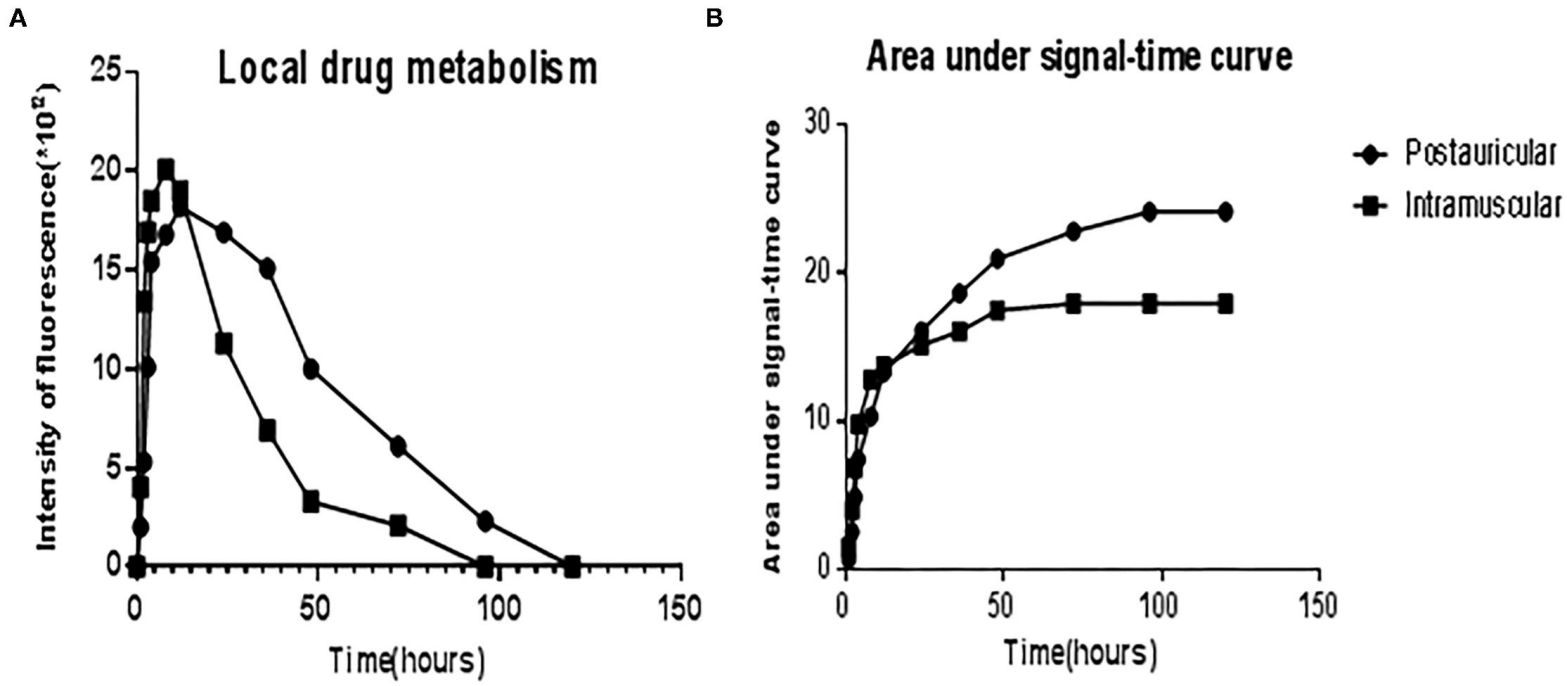
The curve showed the fluorescence intensities of local drug metabolism **(A)** and the area under signal-time curve **(B)** between the postauricular and intramuscular injection groups.

The administered Cy5-dex was found to be concentrated in the local area, and then spread to surrounding tissues after postauricular or intramuscular injections. Intensities of fluorescence in the postauricular and intramuscular injection groups were measured dynamically (Figure 1). The fluorescent intensity reached the peak at 8 h after intramuscular injection, earlier than that by postauricular injection which peaked at 12 h. After the peak-hour, the drug fluorescence in the two groups decreased gradually. The fluorescence in the postauricular injection group decreased at a slower pace than that in the intramuscular injection group. At 96 h post-injection, the drug fluorescence was still present in the postauricular injection group, but undetectable in the intramuscular injection group. The curve for animals receiving postauricular injection showed that the fluorescence dropped slower and steadier than those receiving intramuscular injection (Figure 2A). The area under the metabolic curve of the postauricular group was significantly larger than that of the intramuscular group (*P* < 0.05; Figure 2B).

**Figure 3:**
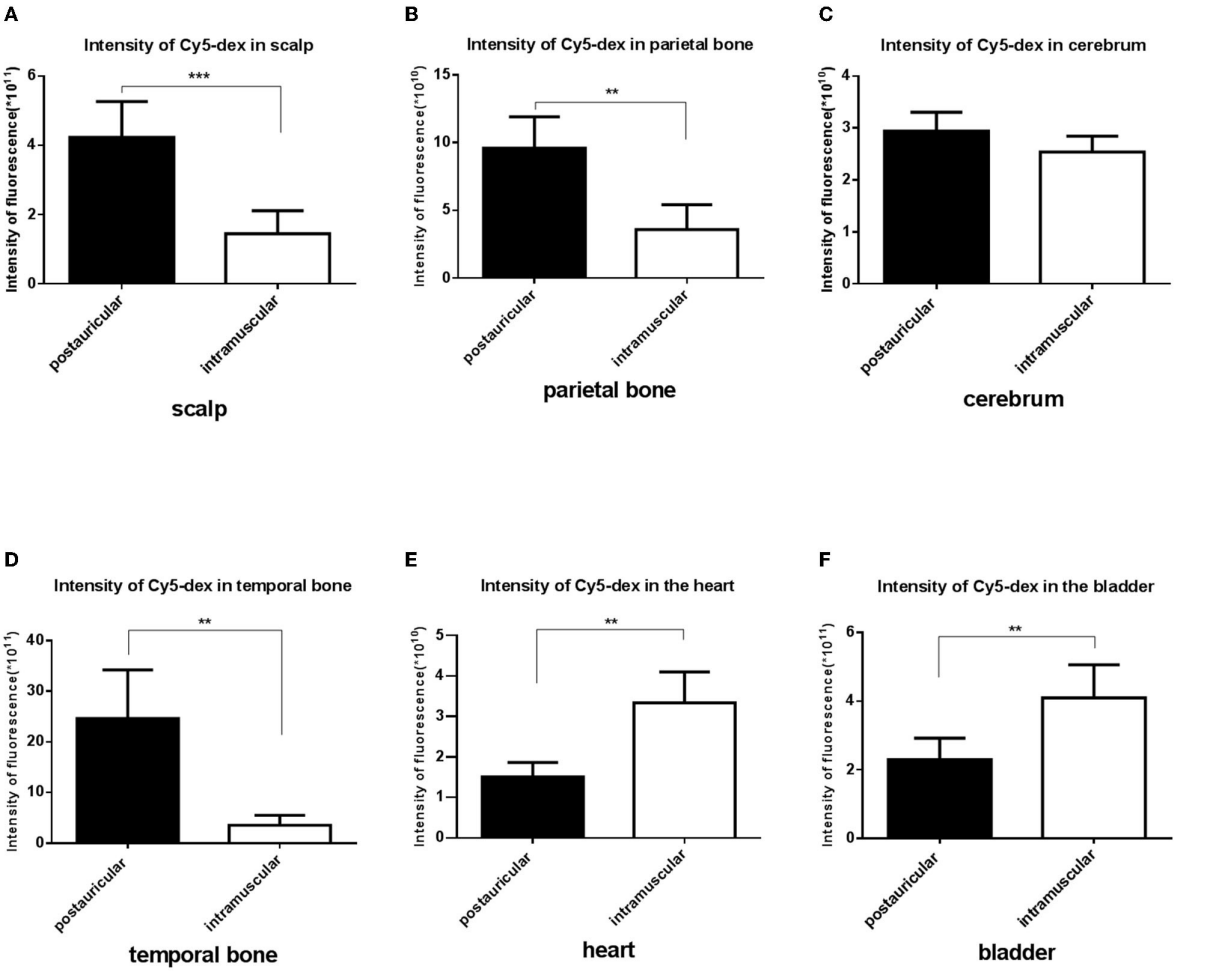
Comparison of the fluorescent intensities of Cy5-dex between the postauricular and intramuscular injection groups in scalp **(A)**, parietal bone **(B)**, cerebrum **(C)**, temporal bone **(D)**, heart **(E)**, and bladder **(F)**. **P < 0.05; ***P < 0.01.

**Figure 4:**
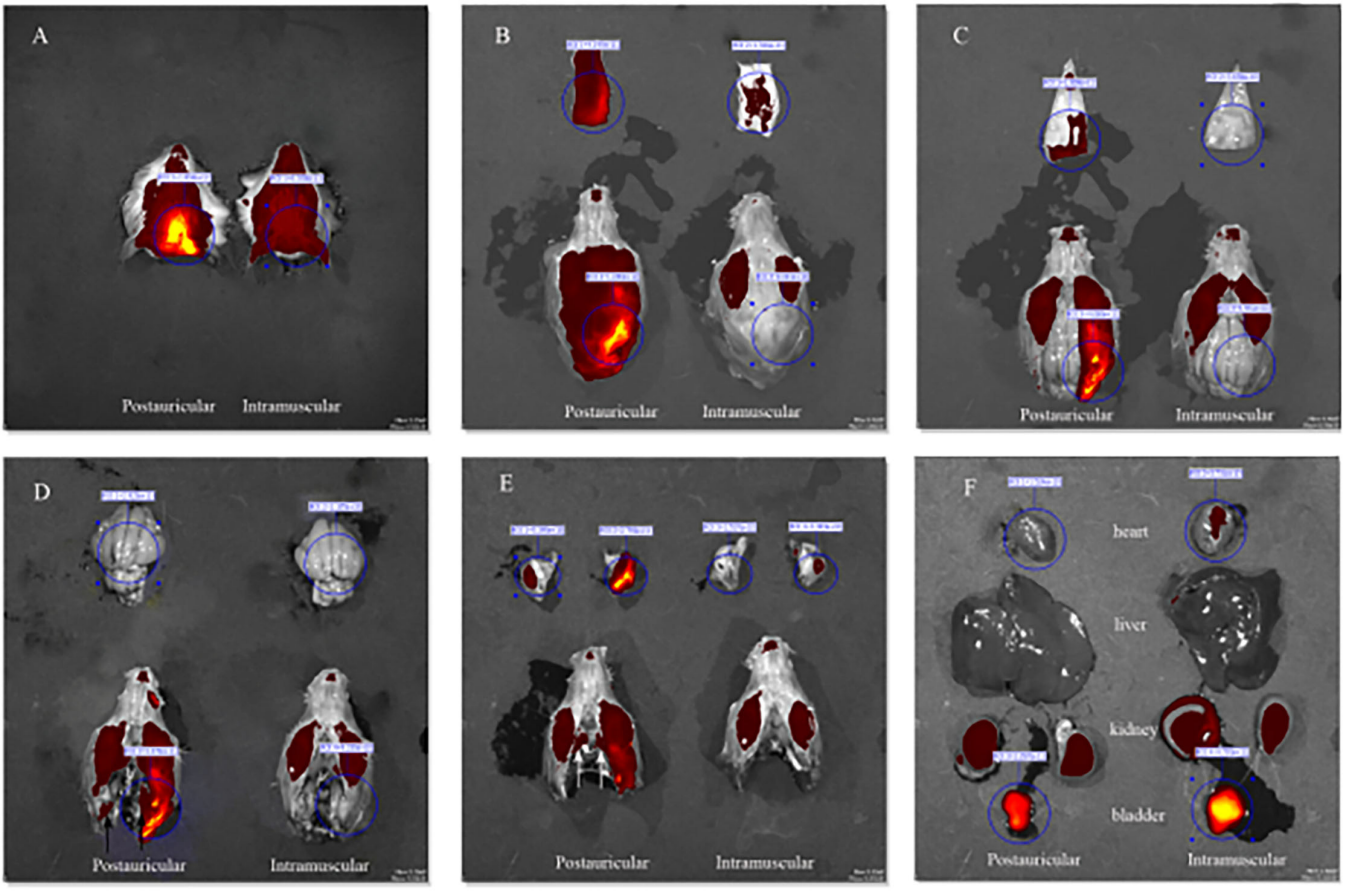
Drug distribution in the head **(A)**, the scalp **(B)**, parietal bone **(C)**, cerebrum (**D**, black arrow, sigmoid sinus), temporal bone (**E**, white arrow, cavernous sinus), and internal organs **(F)**.

Fluorescence intensities in the scalp, parietal bone and temporal bone of the postauricular group were significantly higher than those in the intramuscular group (*P* < 0.01 for each; Figures 3A,B,D). There was no significant difference in the cerebrum between the two groups (*P* > 0.05; Figure 3C). And the fluorescence in the heart and bladder was higher in the intramuscular injection group than the postauricular injection group (*P* < 0.01 for each; Figures 3E,F). Drug distribution in the head and internal organs were shown in Figure 4. The fluorescence in the sigmoid sinus (Figure 4D) and cavernous sinus (Figure 4E) was only visualized in the postauricular injection group, while the fluorescence signals in the kidney and liver were similar between the two groups (Figure 4F).

### Temporal Immunofluorescence

**Figure 5:**
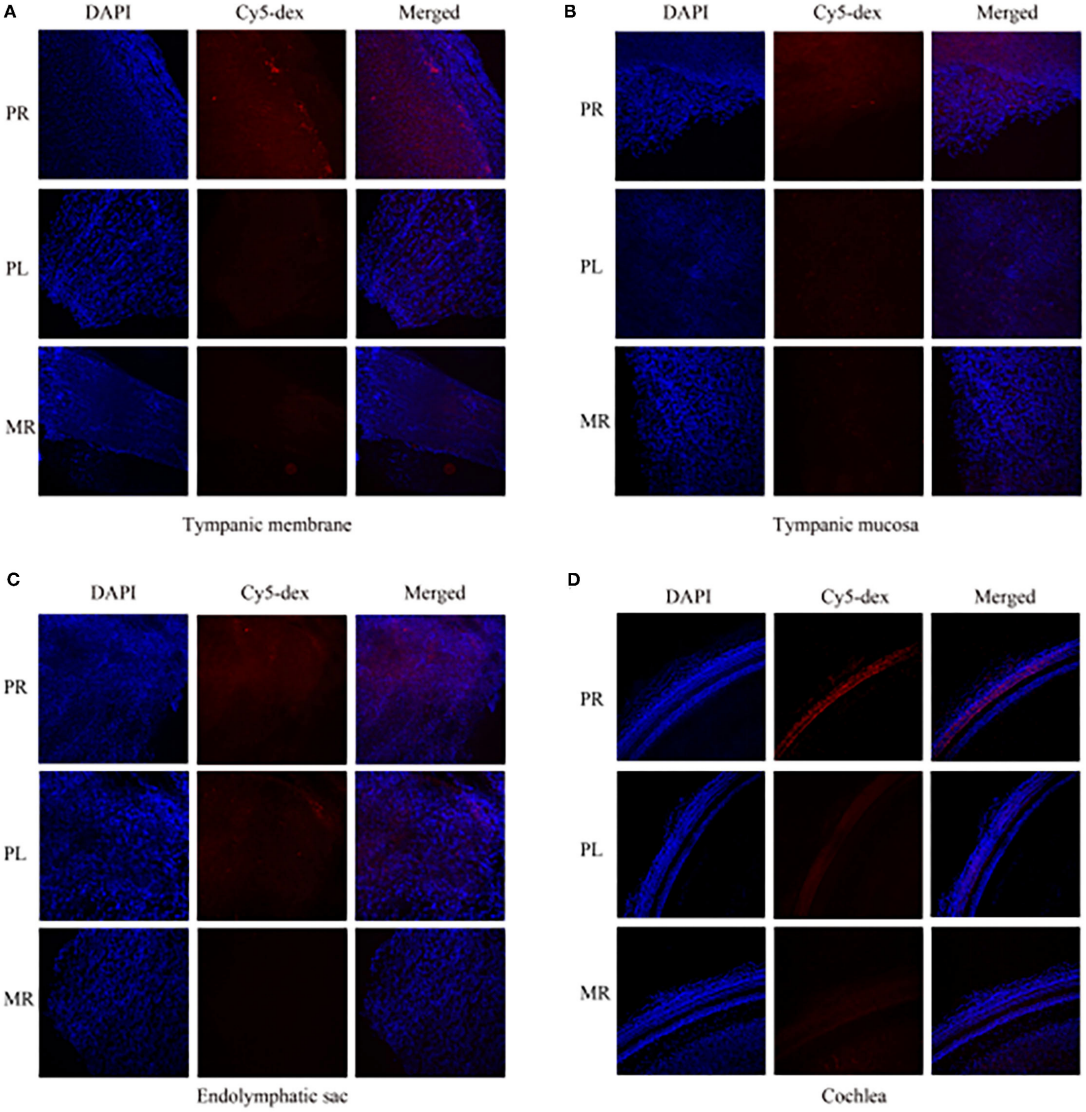
The fluorescent intensities in the tympanic membrane **(A)**, tympanic mucosa **(B)**, endolymphatic sac **(C)**, and cochlea **(D)**. PR, the right tissue of postauricular injection; PL, the left tissue of postauricular injection; MR, the right tissue of intramuscular injection.

**Figure 6:**
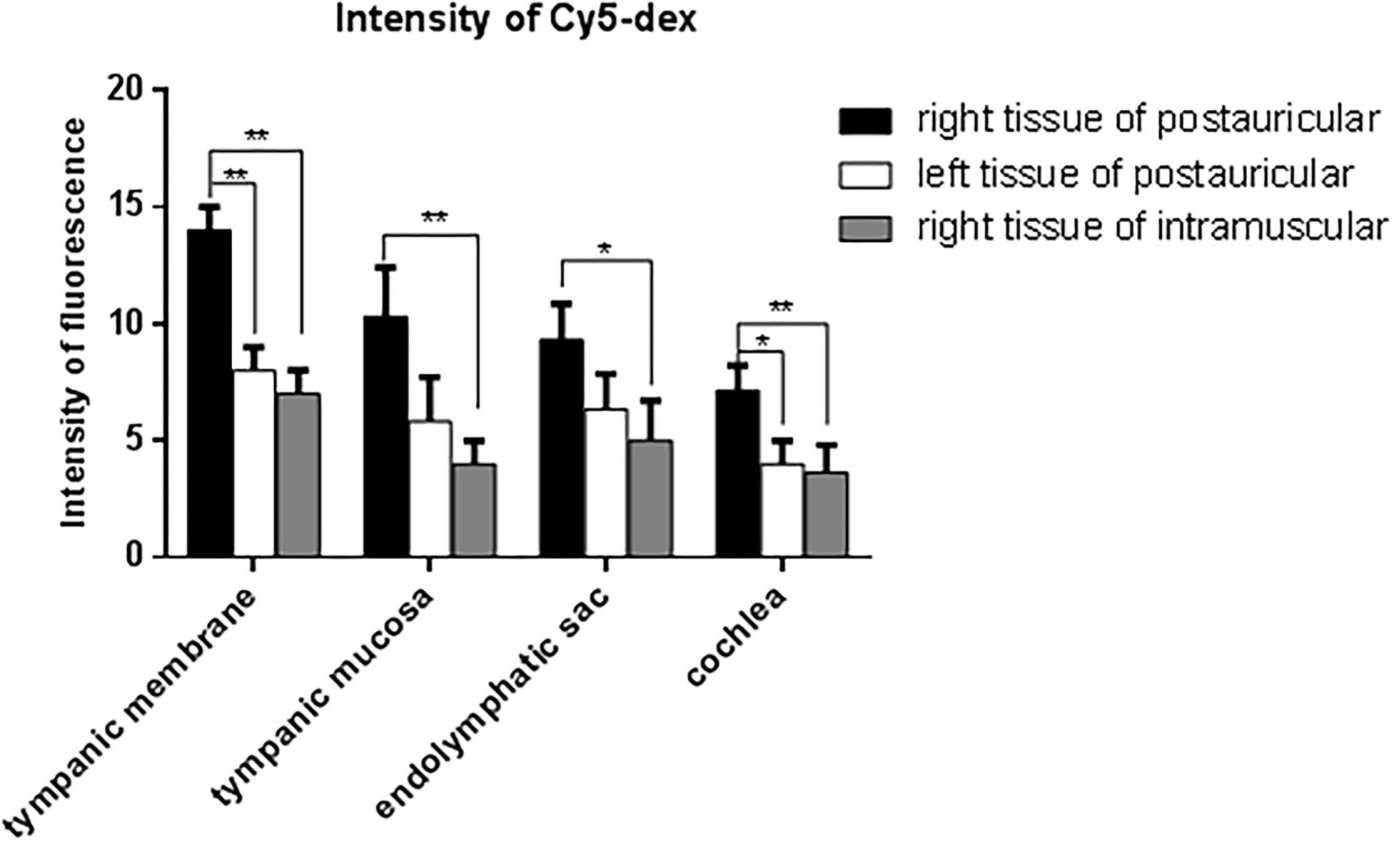
Comparison of fluorescent intensities of Cy5-dex in the tympanic membrane, tympanic mucosa, endolymphatic sac, and cochlea. *P < 0.05. **P < 0.01.

The drug distribution in the tympanic membrane, tympanic mucosa, endolymphatic sac and cochlea of the two groups were generally similar (Figure 5). The fluorescence intensities in these tissues were as follows in descending order: the right side (injection side) of tissues in the postauricular injection group > the left side (non-injection side) of tissues in the postauricular injection group > the right side of tissues in the intramuscular injection group. There were significant differences in the injection sides (right) of tissues between the postauricular and intramuscular injection groups (all *P* < 0.05, Figure 6). As for the tympanic membrane and cochlea in the postauricular injection group, the fluorescence intensity in the right side was significantly higher than that in the left side (both *P* < 0.05). There was no statistical difference in the tympanic mucosa or endolymphatic sac between the right and left sides in the postauricular injection group (both *P* > 0.05). The fluorescence in the cochlea (injection side) of the postauricular injection group was strongest in the basal turn and became weaker from the basal to apical turn, and eventually negligible in the apical turn. Moreover, the spiral artery was visualized in the cochlea (injection side) of the postauricular injection group.

## Discussion

The efficacy of drugs in the inner ear was influenced by the peak concentration, time to peak, areas under concentration-time curve (AUC) etc. (12). Studies on clinical operations and animal anatomy have discovered that the subcutaneous tissues in the postauricular regions are scarce and close to the bone surface, which could result in slower drug absorption by postauricular injection. In our study, we used an *in vivo* optical imaging system to evaluate the pharmacokinetic changes over time (13, 14). The findings showed that the drugs concentrated in the local region for a longer time and decreased slower when postauricularly injected than intramuscularly, which is similar to previous study. The fluorescent intensity in the postauricular injection group was significantly higher than that in the intramuscular injection group. At 96 h post-injection, the drug fluorescence was still strong in the postauricular injection group, while undetectable in the intramuscular injection group. The AUC in the postauricular injection group was significantly larger than that in the intramuscular injection group. The sustained release effect of postauricular injection was obvious, which might result in high drug concentration in the inner ear. In addition, we assessed the drug delivery and distribution in internal organs, and found that drug fluorescence in the heart and bladder of the postauricular injection group was significantly lower than that of the intramuscular injection group. Clinical application also confirmed that glucocorticoid administration *via* postauricular injection showed less adverse effect on blood pressure and blood glucose (15).

The scalp has a rich vascular supply. Many blood vessels, such as the posterior auricular, superficial temporal, occipital, supraorbital and supratrochlear arteries and veins, are woven into a complex meshwork connecting both sides of the scalp. Drugs could be absorbed by the scalp and subcutaneous tissues and reach the contra lateral side. We found the fluorescence intensity in the scalp after postauricular injection was significantly higher than that after intramuscular injection. And high fluorescence intensity in the non-injection side of the tympanic mucosa could also be observed in the postauricular injection animals. The diploe of cranial bones is supplied by the meningeal artery and branches of the scalp arteries. The diploic veins are woven into a complex meshwork and could mutually connect. Among them, the posterior temporal diploic vein goes down to connect with the mastoid emissary vein. Drugs by postauricular injection may enter the diploe of cranial bones through the scalp vascular meshwork and the communication of veins. Compared to intramuscular injection, stronger fluorescence in the diploe by postauricular injection was observed in our experiment. In addition, the fluorescence intensity in the tympanic membrane and tympanic mucosa of injection side in the postauricular injection group was stronger than that of the intramuscular injection group. There are some natural fissures between the post ear and middle ear, such as tympanomastoid fissure and cribriform area, etc. After postauricular injection, drugs could enter the middle ear through these natural fissures and enter the scala tympani through the round window membrane by the local diffusion and permeation pathway (16).

The arterial supply of human endolymphatic sac is mainly derived from the occipital artery and its branches which undergo the mastoid process (17). It's possible that drugs permeates into the occipital artery and then reaches the endolymphatic sac. The present study showed that the fluorescence intensities in the endolymphatic sac and sigmoid sinus were stronger by postauricular injection than those by intramuscular injection. We also found that the mastoid emissary vein of guinea pigs was quite bulky and drained to the sigmoid sinus crossing the mastoid process. Drugs might enter the ipsilateral sigmoid sinus through the mastoid emissary vein. And some of the scalp veins drain into the intracranial venous sinus through the diploic veins. Intracranial venous sinus communicated with each other, and the bilateral sigmoid sinuses were visualized in the excised skull specimens with the *in vivo* optical imaging system. We found that the drug concentration in the bilateral sigmoid sinus of postauricular injection was higher than that of intramuscular injection. The extensive vascular system around the endolymphatic sac consists of both arteries and veins, as well as lymphatic vessels, that are in close contact with the sigmoid sinus (18). Drugs may enter the endolymphatic sac through these connections (19). Therefore, it's likely that drugs by postauricular injection might enter the endolymphatic sac through the occipital artery and connections between the sigmoid sinus and endolymphatic sac.

Drug could be delivered to systemic circulation by postauricular injection, although postauricular subcutaneous tissue was rare and drugs were absorbed slowly. The systemic circulation pathway might cause drugs to permeate into the posterior facial vein through the posterior auricular vein and then drain into the internal jugular vein. Drugs may also enter the sigmoid sinus through the mastoid emissary vein and drain into the internal jugular vein. As the drugs enter systemic circulation, they could reach the Willis Circle through the internal carotid artery and the vertebral artery, and enter the inner ear through the labyrinth artery which is derived from the anterior inferior cerebellar artery. The stylomastoid artery derives from the posterior auricular artery and its terminal blood supplies the inner ear. Drugs *via* postauricular injection may reach the inner ear through the postauricular artery and stylomastoid artery. The artery supplying for facial nerves is reported to be the stylomastoid artery (20). Therefore, our findings confirmed the assumption that facial nerve diseases may be treated by postauricular injection, which warrants further research.

## Conclusion

We evaluated the drug delivery and distribution over time by the *in vivo* optical imaging system and confocal laser scanning microscopy in guinea pigs after postauricular and intramuscular injections, to compare their pharmacokinetic changes. The findings showed that, more drugs concentrated in the inner ear for longer therapeutic time, and less systemic delivery implied less risk of side effects through postauricular injection than intramuscular injection. And the drugs by postauricular injection might enter the endolymphatic sac through the occipital artery and connections between the sigmoid sinus and endolymphatic sac. This study provided a rational support for the local therapy of postauricular injection, which could be more effective and safer than systemic administration for the therapy of inner ear diseases.

## Data Availability Statement

The original contributions presented in the study are included in the article/Supplementary Material, further inquiries can be directed to the corresponding author/s.

## Ethics Statement

The animal study was reviewed and approved by the Animal Care and Use Committee of Shandong Institute of Otolaryngology, Shandong Provincial ENT Hospital.

## Author Contributions

MW, ZF, and HW: conceptualization. WL: data curation. LX: formal analysis. ZH, HW, and MW: funding acquisition. AC and LX: investigation. AC, WL, and ZH: methodology. HW: project administration. MW: supervision. AC and MW: writing—original draft. AC, WL, LX, ZH, ZF, HW, and MW: writing—review and editing. All authors contributed to the article and approved the submitted version.

## Funding

This study was supported by grants from the National Natural Science Foundation of China (81670932 and 81800906), Key Technology Research and Development Program of Shandong (2019GSF108248), and Taishan Scholars Program of Shandong Province (ts20130913).

## Conflict of Interest

The authors declare that the research was conducted in the absence of any commercial or financial relationships that could be construed as a potential conflict of interest.

## Publisher's Note

All claims expressed in this article are solely those of the authors and do not necessarily represent those of their affiliated organizations, or those of the publisher, the editors and the reviewers. Any product that may be evaluated in this article, or claim that may be made by its manufacturer, is not guaranteed or endorsed by the publisher.
